# Crystal structure of (1*R*,2*S*,4*R*,7*R*,8*S*,9*R*)-3,3-dichloro-8,9-epoxy-4,8,12,12-tetramethyltricyclo[5.5.0.0^2,4^]dodecane

**DOI:** 10.1107/S205698901501244X

**Published:** 2015-07-04

**Authors:** Ahmed Benzalim, Aziz Auhmani, My Youssef Ait Itto, Jean-Claude Daran, Abdelwahed Auhmani

**Affiliations:** a Laboratoire de Physico-Chimie Moléculaire et Synthèse Organique, Département de Chimie, Faculté des Sciences Semlalia, BP 2390, Marrakech 40001, Morocco; b Laboratoire de Chimie de Coordination, 205 route de Narbonne, 31077 Toulouse, Cedex 04, France

**Keywords:** crystal structure, absolute configuration, natural products, epoxide

## Abstract

The title compound, C_16_H_24_Cl_2_O, is built up from two fused six- and seven-membered rings which bear a di­chloro­cyclo­propane group and an ep­oxy group, respectively. In the mol­ecule, the six-membered ring adopts an envelope configuration with the C atom linking the ep­oxy ring at the flap, while the seven-membered ring adopts a boat–sofa conformation.

## Related literature   

For applications of epoxides, see: Qu *et al.* (2009 ▸); Taylor *et al.* (1991 ▸); Mori (1989 ▸); Paddon-Jones *et al.* (1997 ▸); Yang (2004 ▸); Vollhardt & Schore (1996 ▸); Trost *et al.* (1983 ▸). For related structures, see: Chiaroni *et al.* (1992 ▸, 1995 ▸, 1996*a* ▸,*b* ▸,*c* ▸); Sbai *et al.* (2002 ▸); Benharref *et al.* (2010 ▸); Oukhrib *et al.* (2013 ▸); Bimoussa *et al.* (2014 ▸). For puckering parameters and ring conformation, see: Boessenkool & Boeyens (1980 ▸).
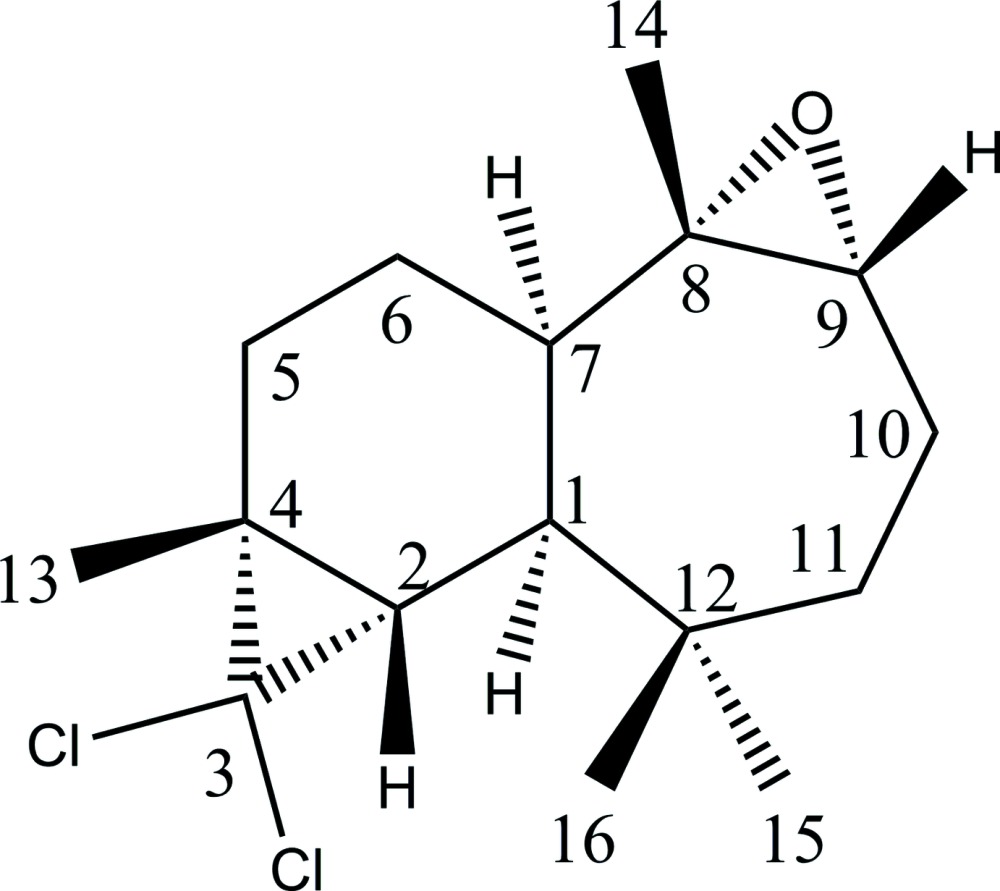


## Experimental   

### Crystal data  

C_16_H_24_Cl_2_O
*M* *_r_* = 303.25
Monoclinic, 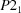

*a* = 8.7706 (5) Å
*b* = 10.5467 (4) Å
*c* = 9.1639 (5) Å
β = 115.710 (7)°
*V* = 763.75 (8) Å^3^
*Z* = 2
Mo *K*α radiation
μ = 0.42 mm^−1^
*T* = 180 K
0.40 × 0.34 × 0.08 mm

### Data collection  

Agilent Xcalibur, Eos, Gemini ultra diffractometer
Absorption correction: multi-scan (*CrysAlis PRO*; Agilent, 2014 ▸) *T*_min_ = 0.901, *T*_max_ = 1.000
7805 measured reflections
2945 independent reflections
2868 reflections with *I* > 2σ(*I*)
*R*_int_ = 0.021

### Refinement  

*R*[*F* ^2^ > 2σ(*F* ^2^)] = 0.029
*wR*(*F* ^2^) = 0.076
*S* = 1.05
2945 reflections
176 parameters
1 restraint
H-atom parameters constrained
Δρ_max_ = 0.44 e Å^−3^
Δρ_min_ = −0.18 e Å^−3^
Absolute structure: Flack *x* determined using 1242 quotients [(*I* ^+^)−(*I* ^−^)]/[(*I* ^+^)+(*I* ^−^)] (Parsons *et al.*, 2013 ▸)
Absolute structure parameter: −0.02 (2)

### 

Data collection: *CrysAlis PRO* (Agilent, 2014 ▸); cell refinement: *CrysAlis PRO*; data reduction: *CrysAlis PRO*; program(s) used to solve structure: *SIR97* (Altomare *et al.*, 1999 ▸); program(s) used to refine structure: *SHELXL2013* (Sheldrick, 2015 ▸); molecular graphics: *ORTEPIII* (Burnett & Johnson, 1996 ▸) and *ORTEP-3 for Windows* (Farrugia, 2012 ▸); software used to prepare material for publication: *SHELXL2013*.

## Supplementary Material

Crystal structure: contains datablock(s) I, global. DOI: 10.1107/S205698901501244X/xu5855sup1.cif

Structure factors: contains datablock(s) I. DOI: 10.1107/S205698901501244X/xu5855Isup2.hkl

Supporting information file. DOI: 10.1107/S205698901501244X/xu5855Isup3.cml

**Fig. 1.**
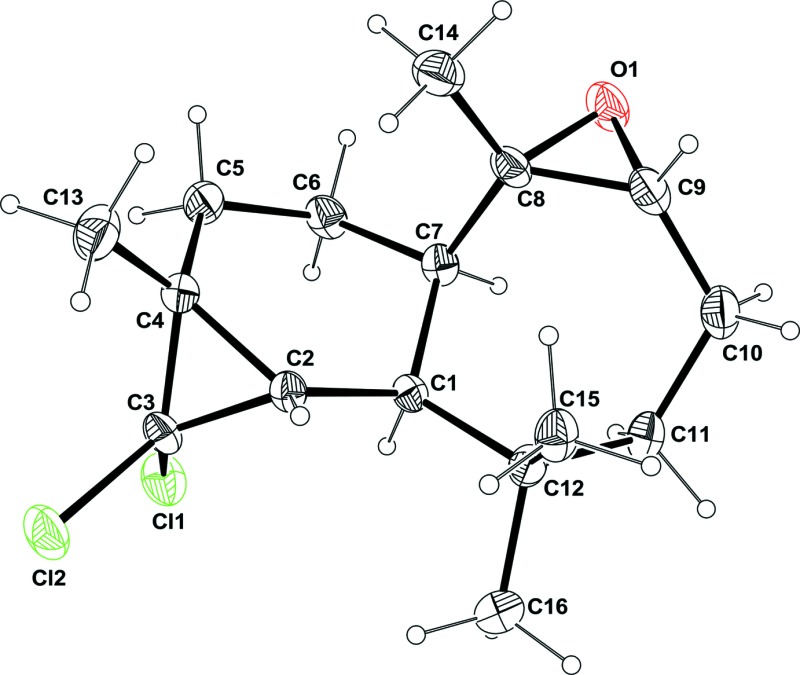
Displacement ellipsoid plot of the title compound.

. DOI: 10.1107/S205698901501244X/xu5855fig1.tif
Displacement ellipsoid plot of the title compound.

CCDC reference: 1409393

Additional supporting information:  crystallographic information; 3D view; checkCIF report

## supplementary crystallographic information

### S1. Chemical context

Epoxides are valuable inter­mediates frequently used as versatile building blocks in organic synthesis (Qu *et al.*, 2009). Thus, epoxides are important precursors in the synthesis of Anti­fungal products (Taylor *et al.*, 1991) and different pheromones (Mori, 1989, Paddon-Jones *et al.*, 1997). Besides, many natural products possess this functional group as an essential structural moiety for their biological activities (Yang, 2004; Vollhardt & Schore, 1996; Trost *et al.*, 1983). Because of their widespread occurrence, biological and synthetic utilities, the synthesis of new epoxides has grown significantly.

In the aim of preparing new epoxides from natural products, we recently synthetise γ-Ep­oxy­himachalene 1 (scheme 1) from naturally occurred sesquiterpene γ-himachalene without crystallographic evidence of its absolute configuration as the product was oily. We therefore decided to transform it into a solid derivative by [2+1] cyclo­addition reaction of a dihalocarbene on the remaining cyclo­hexenic double bond.

The structure of the newly prepared 2 (scheme 2) has been established from its ^1^H and ^13^C NMR spectral data. An X-ray structure analysis has allowed us to determine unambiguously its stereochemistry and deduce the absolute configuration of its oily precursor γ-Ep­oxy­himachalene 1.

### S2. Structural commentary

Compound 2 is built up from two fused 6 and 7 membered rings (Fig. 1). The seven membered ring is bearing an ep­oxy group whereas the 6 membered ring bears a di­chloro­cyclo­propane. In the seven membered ring, the puckering parameters Q2= 0.9692 (15), Q2= 0.2716 (52) and φ2= 97.11, φ3= 74.34 agree with a boat sofa conformation (Boessenkool & Boeyens, 1980). The six membered ring displays an envelope conformation with the puckering parameters θ = 125.90° and φ2 = 118.89° (Cremer & Pople, 1975).

### S3. Database survey

A search in the Cambridge Structural Database, version 5.36 reveals 9 hits with related structure having two fused 6 and 7 membered rings (Chiaroni *et al.*, 1992, 1995; Chiaroni *et al.*, 1996*a*,b,c; Sbai *et al.*, 2002; Benharref *et al.*, 2010; Oukhrib *et al.*, 2013; Bimoussa *et al.*, 2014 )

### S4. Synthesis and crystallization

Thus, the di­chloro­carbene, generated at 0°C from an excess of CHCl_3_ (0,93 mL, 11,59 mmol) and solid t-BuOK (1,3 g, 11,58 mmol), reacts in the presence of tri­ethyl­benzyl­ammonium chloride (100 mg, 0.439 mmol) as catalyst, with γ-Ep­oxy­himachalene 1 (0,650 g, 2,95 mmol) to give 22% yield (200 mg) of the cyclo­adduct C_16_H_24_OCl_2_2.

### S5. Refinement

Crystal data, data collection and structure refinement details are summarized in Table 1. All H atoms attached to C atoms were fixed geometrically and treated as riding with C—H = 0.99 Å (methyl­ene), 0.98 Å (methyl), 0.95Å (methine) with U_iso_(H) = 1.2U_eq_(CH and CH_2_) or U_iso_(H) = 1.5U_eq_(CH_3_).

### Crystal data

**Table:**

C_16_H_24_Cl_2_O	*F*(000) = 324
*M**_r_* = 303.25	*D*_x_ = 1.319 Mg m^−^^3^
Monoclinic, *P*2_1_	Mo *K*α radiation, λ = 0.71073 Å
*a* = 8.7706 (5) Å	Cell parameters from 4267 reflections
*b* = 10.5467 (4) Å	θ = 4.3–29.3°
*c* = 9.1639 (5) Å	µ = 0.42 mm^−^^1^
β = 115.710 (7)°	*T* = 180 K
*V* = 763.75 (8) Å^3^	Box, colourless
*Z* = 2	0.40 × 0.34 × 0.08 mm

### Data collection

**Table:**

Agilent Xcalibur, Eos, Gemini ultra diffractometer	2945 independent reflections
Radiation source: Enhance (Mo) X-ray Source	2868 reflections with *I* > 2σ(*I*)
Graphite monochromator	*R*_int_ = 0.021
Detector resolution: 16.1978 pixels mm^-1^	θ_max_ = 26.4°, θ_min_ = 3.2°
ω scans	*h* = −10→10
Absorption correction: multi-scan (*CrysAlis PRO*; Agilent, 2014)	*k* = −13→12
*T*_min_ = 0.901, *T*_max_ = 1.000	*l* = −11→11
7805 measured reflections	

### Refinement

**Table:**

Refinement on *F*^2^	Hydrogen site location: inferred from neighbouring sites
Least-squares matrix: full	H-atom parameters constrained
*R*[*F*^2^ > 2σ(*F*^2^)] = 0.029	*w* = 1/[σ^2^(*F*_o_^2^) + (0.0453*P*)^2^ + 0.1659*P*] where *P* = (*F*_o_^2^ + 2*F*_c_^2^)/3
*wR*(*F*^2^) = 0.076	(Δ/σ)_max_ < 0.001
*S* = 1.05	Δρ_max_ = 0.44 e Å^−^^3^
2945 reflections	Δρ_min_ = −0.18 e Å^−^^3^
176 parameters	Absolute structure: Flack *x* determined using 1242 quotients [(*I*^+^)-(*I*^-^)]/[(*I*^+^)+(*I*^-^)] (Parsons *et al.*, 2013)
1 restraint	Absolute structure parameter: −0.02 (2)

### Special details

**Table:**

Geometry. All e.s.d.'s (except the e.s.d. in the dihedral angle between two l.s. planes) are estimated using the full covariance matrix. The cell e.s.d.'s are taken into account individually in the estimation of e.s.d.'s in distances, angles and torsion angles; correlations between e.s.d.'s in cell parameters are only used when they are defined by crystal symmetry. An approximate (isotropic) treatment of cell e.s.d.'s is used for estimating e.s.d.'s involving l.s. planes.

### Fractional atomic coordinates and isotropic or equivalent isotropic displacement parameters (Å^2^)

**Table:**

	*x*	*y*	*z*	*U*_iso_*/*U*_eq_	
C1	0.6765 (3)	1.0011 (2)	0.7612 (3)	0.0150 (5)	
H1	0.5811	0.9485	0.6832	0.018*	
C2	0.7132 (3)	1.0959 (2)	0.6554 (3)	0.0161 (5)	
H2	0.7162	1.1866	0.6885	0.019*	
C3	0.6463 (3)	1.0746 (2)	0.4760 (3)	0.0184 (5)	
C4	0.8324 (3)	1.0632 (2)	0.5788 (3)	0.0183 (5)	
C5	0.9157 (3)	0.9334 (3)	0.6122 (3)	0.0238 (5)	
H5A	1.0399	0.9457	0.6641	0.029*	
H5B	0.8860	0.8909	0.5070	0.029*	
C6	0.8699 (3)	0.8432 (3)	0.7196 (3)	0.0222 (5)	
H6A	0.7739	0.7896	0.6480	0.027*	
H6B	0.9675	0.7863	0.7775	0.027*	
C7	0.8220 (3)	0.9058 (2)	0.8456 (3)	0.0175 (5)	
H7	0.7706	0.8360	0.8832	0.021*	
C8	0.9699 (3)	0.9526 (3)	0.9984 (3)	0.0200 (5)	
C9	0.9417 (3)	0.9677 (3)	1.1444 (3)	0.0234 (6)	
H9	1.0197	1.0302	1.2236	0.028*	
C10	0.7742 (3)	0.9537 (3)	1.1534 (3)	0.0266 (6)	
H10A	0.7722	1.0173	1.2319	0.032*	
H10B	0.7739	0.8692	1.2003	0.032*	
C11	0.6069 (3)	0.9666 (3)	0.9990 (3)	0.0230 (6)	
H11A	0.5167	0.9896	1.0317	0.028*	
H11B	0.5778	0.8825	0.9461	0.028*	
C12	0.6043 (3)	1.0642 (2)	0.8722 (3)	0.0189 (5)	
C13	0.9439 (4)	1.1678 (3)	0.5645 (3)	0.0287 (6)	
H13A	0.9746	1.1479	0.4762	0.043*	
H13B	1.0468	1.1748	0.6664	0.043*	
H13C	0.8823	1.2485	0.5416	0.043*	
C14	1.1115 (3)	1.0267 (3)	0.9879 (3)	0.0278 (6)	
H14A	1.1956	1.0487	1.0972	0.042*	
H14B	1.0661	1.1045	0.9255	0.042*	
H14C	1.1651	0.9752	0.9342	0.042*	
C15	0.6951 (3)	1.1861 (2)	0.9559 (3)	0.0222 (5)	
H15A	0.6600	1.2559	0.8774	0.033*	
H15B	0.8178	1.1740	0.9988	0.033*	
H15C	0.6658	1.2065	1.0449	0.033*	
C16	0.4186 (3)	1.0978 (3)	0.7640 (3)	0.0294 (6)	
H16A	0.3545	1.0200	0.7184	0.044*	
H16B	0.4125	1.1533	0.6760	0.044*	
H16C	0.3704	1.1415	0.8287	0.044*	
O1	1.0269 (3)	0.85597 (19)	1.1245 (2)	0.0275 (4)	
Cl1	0.52452 (8)	0.93841 (6)	0.38605 (7)	0.02818 (17)	
Cl2	0.56482 (9)	1.20376 (6)	0.34222 (7)	0.03117 (18)	

### Atomic displacement parameters (Å^2^)

**Table:**

	*U*^11^	*U*^22^	*U*^33^	*U*^12^	*U*^13^	*U*^23^
C1	0.0159 (11)	0.0142 (11)	0.0135 (10)	−0.0016 (9)	0.0052 (9)	−0.0003 (9)
C2	0.0194 (12)	0.0136 (12)	0.0158 (11)	0.0011 (10)	0.0082 (9)	0.0008 (9)
C3	0.0220 (12)	0.0154 (11)	0.0155 (11)	0.0027 (10)	0.0060 (9)	0.0039 (9)
C4	0.0182 (12)	0.0205 (12)	0.0169 (11)	0.0013 (10)	0.0083 (9)	0.0011 (9)
C5	0.0258 (12)	0.0274 (14)	0.0212 (11)	0.0085 (12)	0.0130 (9)	0.0008 (12)
C6	0.0277 (14)	0.0172 (12)	0.0200 (11)	0.0075 (11)	0.0090 (10)	0.0018 (9)
C7	0.0223 (12)	0.0133 (12)	0.0173 (11)	0.0008 (9)	0.0089 (10)	0.0007 (9)
C8	0.0215 (12)	0.0190 (12)	0.0158 (10)	0.0055 (11)	0.0047 (9)	0.0038 (10)
C9	0.0276 (13)	0.0234 (14)	0.0155 (11)	0.0049 (11)	0.0060 (10)	0.0011 (10)
C10	0.0348 (14)	0.0279 (14)	0.0203 (11)	0.0043 (13)	0.0150 (11)	0.0035 (11)
C11	0.0285 (13)	0.0240 (14)	0.0216 (11)	−0.0017 (11)	0.0158 (10)	0.0012 (10)
C12	0.0198 (12)	0.0208 (13)	0.0183 (11)	−0.0004 (10)	0.0102 (9)	−0.0002 (10)
C13	0.0301 (14)	0.0327 (16)	0.0284 (13)	−0.0062 (13)	0.0175 (11)	−0.0007 (12)
C14	0.0215 (13)	0.0320 (15)	0.0251 (13)	−0.0017 (12)	0.0057 (11)	0.0026 (12)
C15	0.0309 (13)	0.0175 (13)	0.0213 (11)	0.0026 (11)	0.0142 (10)	−0.0009 (11)
C16	0.0222 (14)	0.0384 (16)	0.0296 (13)	0.0054 (13)	0.0132 (11)	0.0021 (13)
O1	0.0334 (11)	0.0271 (11)	0.0197 (9)	0.0117 (9)	0.0094 (8)	0.0075 (8)
Cl1	0.0305 (3)	0.0301 (3)	0.0191 (3)	−0.0081 (3)	0.0062 (2)	−0.0044 (3)
Cl2	0.0418 (4)	0.0283 (3)	0.0250 (3)	0.0152 (3)	0.0159 (3)	0.0122 (3)

### Geometric parameters (Å, º)

**Table:**

C1—C2	1.521 (3)	C9—O1	1.449 (3)
C1—C7	1.542 (3)	C9—C10	1.513 (4)
C1—C12	1.561 (3)	C9—H9	1.0000
C1—H1	1.0000	C10—C11	1.540 (4)
C2—C3	1.503 (3)	C10—H10A	0.9900
C2—C4	1.530 (3)	C10—H10B	0.9900
C2—H2	1.0000	C11—C12	1.545 (3)
C3—C4	1.494 (4)	C11—H11A	0.9900
C3—Cl2	1.764 (2)	C11—H11B	0.9900
C3—Cl1	1.766 (3)	C12—C15	1.532 (4)
C4—C13	1.517 (4)	C12—C16	1.536 (4)
C4—C5	1.519 (4)	C13—H13A	0.9800
C5—C6	1.542 (4)	C13—H13B	0.9800
C5—H5A	0.9900	C13—H13C	0.9800
C5—H5B	0.9900	C14—H14A	0.9800
C6—C7	1.538 (3)	C14—H14B	0.9800
C6—H6A	0.9900	C14—H14C	0.9800
C6—H6B	0.9900	C15—H15A	0.9800
C7—C8	1.520 (3)	C15—H15B	0.9800
C7—H7	1.0000	C15—H15C	0.9800
C8—O1	1.457 (3)	C16—H16A	0.9800
C8—C9	1.471 (3)	C16—H16B	0.9800
C8—C14	1.505 (4)	C16—H16C	0.9800
			
C2—C1—C7	112.96 (19)	O1—C9—C10	119.6 (2)
C2—C1—C12	113.1 (2)	C8—C9—C10	126.1 (2)
C7—C1—C12	115.62 (19)	O1—C9—H9	113.6
C2—C1—H1	104.6	C8—C9—H9	113.6
C7—C1—H1	104.6	C10—C9—H9	113.6
C12—C1—H1	104.6	C9—C10—C11	120.1 (2)
C3—C2—C1	120.6 (2)	C9—C10—H10A	107.3
C3—C2—C4	59.00 (16)	C11—C10—H10A	107.3
C1—C2—C4	121.3 (2)	C9—C10—H10B	107.3
C3—C2—H2	114.9	C11—C10—H10B	107.3
C1—C2—H2	114.9	H10A—C10—H10B	106.9
C4—C2—H2	114.9	C10—C11—C12	116.6 (2)
C4—C3—C2	61.39 (16)	C10—C11—H11A	108.2
C4—C3—Cl2	120.18 (19)	C12—C11—H11A	108.2
C2—C3—Cl2	119.53 (18)	C10—C11—H11B	108.2
C4—C3—Cl1	120.21 (18)	C12—C11—H11B	108.2
C2—C3—Cl1	120.14 (18)	H11A—C11—H11B	107.3
Cl2—C3—Cl1	108.85 (13)	C15—C12—C16	107.7 (2)
C3—C4—C13	117.1 (2)	C15—C12—C11	110.4 (2)
C3—C4—C5	119.9 (2)	C16—C12—C11	107.7 (2)
C13—C4—C5	113.6 (2)	C15—C12—C1	114.5 (2)
C3—C4—C2	59.62 (16)	C16—C12—C1	107.1 (2)
C13—C4—C2	118.0 (2)	C11—C12—C1	109.2 (2)
C5—C4—C2	118.4 (2)	C4—C13—H13A	109.5
C4—C5—C6	116.7 (2)	C4—C13—H13B	109.5
C4—C5—H5A	108.1	H13A—C13—H13B	109.5
C6—C5—H5A	108.1	C4—C13—H13C	109.5
C4—C5—H5B	108.1	H13A—C13—H13C	109.5
C6—C5—H5B	108.1	H13B—C13—H13C	109.5
H5A—C5—H5B	107.3	C8—C14—H14A	109.5
C7—C6—C5	116.5 (2)	C8—C14—H14B	109.5
C7—C6—H6A	108.2	H14A—C14—H14B	109.5
C5—C6—H6A	108.2	C8—C14—H14C	109.5
C7—C6—H6B	108.2	H14A—C14—H14C	109.5
C5—C6—H6B	108.2	H14B—C14—H14C	109.5
H6A—C6—H6B	107.3	C12—C15—H15A	109.5
C8—C7—C6	115.4 (2)	C12—C15—H15B	109.5
C8—C7—C1	116.08 (19)	H15A—C15—H15B	109.5
C6—C7—C1	109.83 (19)	C12—C15—H15C	109.5
C8—C7—H7	104.7	H15A—C15—H15C	109.5
C6—C7—H7	104.7	H15B—C15—H15C	109.5
C1—C7—H7	104.7	C12—C16—H16A	109.5
O1—C8—C9	59.31 (15)	C12—C16—H16B	109.5
O1—C8—C14	113.9 (2)	H16A—C16—H16B	109.5
C9—C8—C14	118.1 (2)	C12—C16—H16C	109.5
O1—C8—C7	111.4 (2)	H16A—C16—H16C	109.5
C9—C8—C7	117.4 (2)	H16B—C16—H16C	109.5
C14—C8—C7	120.5 (2)	C9—O1—C8	60.82 (15)
O1—C9—C8	59.88 (15)		
