# Supplementary figures and images for: Crystal structure of (1R,2S,4R,7R,8S,9R)-3,3-dichloro-8,9-epoxy-4,8,12,12-tetramethyltricyclo[5.5.0.02,4]dodecane
Source: Acta Crystallogr E Crystallogr Commun. 2015 Jul 4;71(Pt 8):o538–9. doi: 10.1107/S205698901501244X (PMC4571385; doi:10.1107/S205698901501244X)

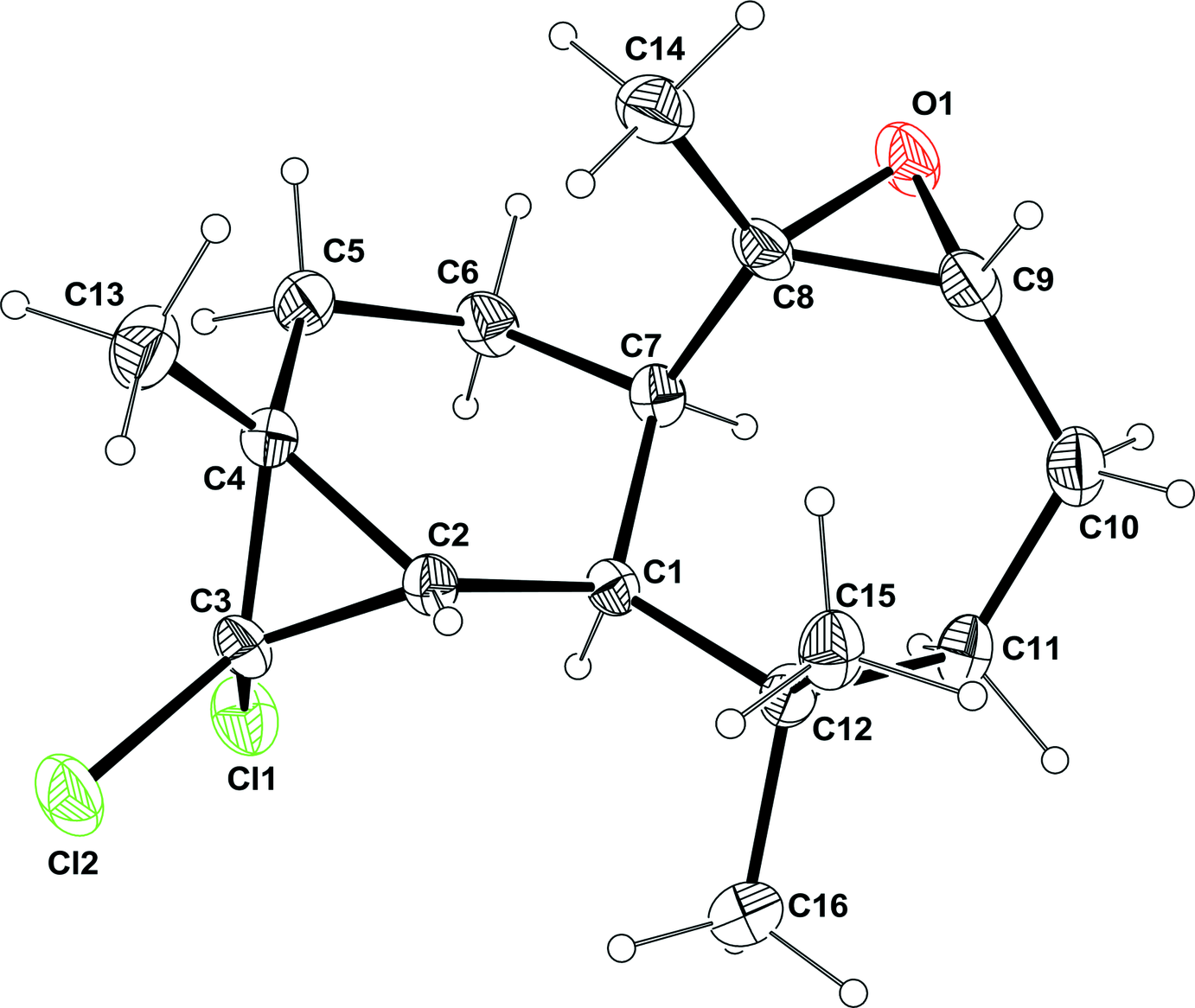

Supplement: Supplementary file 4 [file e-71-0o538-fig1.tif]
